# Physiology

**Published:** 1876-01

**Authors:** 


### VII. PHYSIOLOGY.

1. *A Method of Observing the Circulation in the Frog's Lungs.* F. HOLMGREN. (*Centralbl. f. Chir.*, No. 39, 1875.)

The frog (rana esculenta is the preferable species) is poisoned by several small doses of curare, so as to be paralyzed for two or three days. A broad fold of the skin is taken up near the arm pit, and a curved needle, armed with a silk thread, is carried through the basis of this fold, whereupon the thread is tied. In the same manner a ligature is applied to the skin near the hind legs. Between both ligatures a sufficient portion of the skin and the thin muscular layer is removed, when the inflated lung will protrude through the wound, and soon collapse.

The frog securely fastened upon a board in the well-known manner, the lung is put into a chamber which fits over the hole in the table of the microscope, and is closed at both ends by glass, to allow the light to pass from the reflector through the chamber into the tube of the microscope. If now the lung is inflated again through a rubber tube, a most beautiful view of the circulation can be witnessed.

2. *The Vaso-Motor Nerves and their Action.* PROFESSORS MASIUS AND VANLAIR. (*Le Progrès Médical*, Oct. 2, 1875.)

1st. The vaso-motor nerves are a part of the vegetative nervous system; they have their principal origin in the spinal cord and medulla oblongata; they arise, accessorily, from the sub-medullary portion of the encephalon, from the ganglions of the sympathetic situated upon the cord, and are distributed to the periphery along the course of the nervous fibres.

2nd. In passing from the medullary axis to the lateral roots, the vaso-motor nerves pass along the anterior branches; they pass along to the vessels, either uniting themselves with the spinal and cranial nerves, or accompanying the arteries.

3rd. The vaso-motor filaments are distributed to the muscular coat of the vessels, and form, at their termination, numerous plexuses, provided with microscopic ganglions.

It is not certain that the nerve fibres penetrate the interior of the cellules which constitute the muscular tunic.

4th. The influence exercised by the vaso-motor nerves upon the calibre of the vessels is incontestible. Of these nerves, some produce, when irritated, contraction of the vessels which they supply; others, on the contrary, produce, upon excitation, a dilating effect.

5th. The vaso-constrictor fibres and the vaso-dilator fibres are probably united in one nerve, in such a manner that the action provoked by an excitant can differ according to the predominance of one or the other species of fibres.

6th. The vaso-motor nerves are dependent upon the centres from which they arise, and exhibit their action by their transmitting it.

The activity of the centres may be direct or reflex, and gives rise to vaso-constrictor or vaso-dilator effects.

7th. The existence of terminal nervous apparatuses, situated in the vascular walls, must be admitted. They are composed of the microscopic ganglions, distributed among the plexuses which terminate the vaso-motor nerves.

These ganglions are the small *tonic* vaso-motor centres.

8th. The vaso-dilator nerves have for their function the moderating of the constrictor power of the latter centres, and the augmenting thereby of the calibre of the vessels.

9th. The vaso-motor nerves, in their course across the medulla, are distributed to the side from which they arise.

The influence of the parts of the encephalon situated in front of the tubercula quadrigemina is transmitted to the opposite side.

10th. The vaso-motor nerves, by virtue of their action upon the calibre of the vessels, have not only the power of modifying the rapidity of the flow of the blood, but they act also upon the vascular tension, as well as upon the temperature, the color and the composition of the blood.

They are equally concerned in the phenomena of absorption, nutrition and secretion.

